# Cultural Capital and High Risk Behaviors Among Youth

**DOI:** 10.5812/ijhrba.17595

**Published:** 2014-03-10

**Authors:** Golnar Dehghan Dehnavi, Mehraban Parsamehr, Samaneh Naseri

**Affiliations:** 1 Department of Sociology, Yazd University, Yazd, IR Iran; 2 Department of Sociology, Kharazmi University, Tehran, IR Iran

**Keywords:** High-Risk Behaviors, Cultural Capital, Students

*Dear Editor,*

High risk behaviors increase the likelihood of destructive physical, psychological and social consequences for the individual. These behaviors are evident among adolescents who are extremely dependent on each other and follow their peers’ patterns. Moreover, sexual risk-taking has been recently highlighted as a reason for unplanned pregnancy, infectious diseases and HIV (1).

Cultural capital, a sociological term, has been extensively used since its introduction by Bourdieu in 1973. According to him, cultural capital consists of knowledge, skills, education and advantages an individual that allows him/her to attain a higher social status (2).

Yazd University with a total of almost 10000 students was selected as the study population and a sample was chosen via proportionate classification method. In this method, the number of subjects in each major based on total number of subjects in that major was randomly selected. The formula for sample calculation is as follows: 

[n = (N × t^2^ × p × q) / (N × d^2^+ t^2^ × p × q)],

Where n denotes sample size, N refers to population size, p refers to proportion of the population with certain characteristics, q refers to proportion of the population lacks certain characteristics, d refers to confidence interval, and t refer to confidence level respectively.

To collect the required data, a questionnaire including 6 demographic items (researcher- made), 15 cultural capital items (researcher–made), and 33 items (standard) related to high risk behaviors was used. To analyze the data, SPSS software version 16 was applied. Chronbach’s alpha, an index for measurement of internal reliability, achieved 0.75, which shows a reasonable rate of reliability for all items when α = .05. Items of the questionnaire assessed three states of cultural capital; the embodied state (reading book, papers, magazines, use of the internet, and knowing a foreign language), the objectified state (possessing written cultural products, audio and video products and cultural instruments), and the institutionalized state(a series of educational certificates, attending art, sport and religious classes and seminars), and their association with high risk behaviors (drugs addiction, smoking, alcohol use, aggression, opposite sex relation and dangerous driving).

To examine the cultural capital association with the incidence of high risk behaviors, the Pearson’s correlation coefficient was used. The coefficient correlation value was equal to -0.255 when significant level was at 0.000. So, there was a negative and weak correlation between cultural capital and the incidence of high risk behaviors and since the level of significance is lower than 0.05, thus the correlation is confirmed.

Considering the results, the mean score for high risk behaviors are 145.61 and 164.71 for boys and girls respectively. So, the higher boys’ score than girls’, showed their more willingness toward high risk behaviors. These finding are similar to researches of Nazemi (3), Tavakolizad (4), Bachoo (5), and their colleagues.

Furthermore, the students’ age are negatively correlated with the incidence rate of high risk behaviors. That is, as students become older, the rate of high risk behaviors decreases. These findings are also similar to researches of Ghodsi (6), Sohrabi (7), Kaldi and Falahminbashi (8) and their colleagues.

No meaningful correlation was observed between cultural capital and sex, or age. The average rate of high risk behaviors among different scientific departments were as follows: humanities 162.7, science 153.7, mathematics 147.0, engineering 142.9, natural resources and desert studies 174.3, and art and architecture 145.2. Considering the level of significance, the field of study and high risk behaviors was meaningful and this rate was higher among students of natural resources and desert studies compare to other majors. Moreover, a negative relationship was identified between the rate of cultural capital and the incidence of high risk behaviors and also between embodied and institutionalized cultural capital and high risk behaviors. However, no meaningful association was observed between objectified cultural capital and high risk behaviors.

The embodied cultural capital is personal and consists of a set of competencies and skills the individual acquires; therefore, achieving such cultural capital takes some time and trainings. This capital is neither inherited, nor can be bought. However, the objectified cultural capital includes material belongings, which could be transferred from person to person. For its measurement, total properties and cultural goods used by the individual should be taken into consideration that indicates the person’s willingness in consuming cultural products. The present research results show that possession of cultural products and instruments does not meaningfully associate with a decrease in individual’s tendency towards high risk behaviors. But, increasing intrinsic awareness, competencies and skills (which are acquired by training and practice and cannot be transferred to others), can significantly minimize the individual tendency towards high risk behaviors.

Thus, it is recommended for youths and students to maximize the rate of cultural capital through applying the mentioned strategies. Then, as the level of their cultural capital goes up, their behaviors and habits will be shaped and degree of risk taking and tendency to high risk behaviors will definitely decrease.
